# Systematic Evaluation of Systemic Right Ventricular Function

**DOI:** 10.3390/jcm9010107

**Published:** 2019-12-31

**Authors:** Matthias Schneider, Matthias Beichl, Christian Nietsche, Dietrich Beitzke, Gerold Porenta, Gilbert Beran, Karin Vonbank, Jakob Hauser, Christian Hengstenberg, Georg Goliasch, Thomas Binder, Harald Gabriel

**Affiliations:** 1 Department of Internal Medicine II, cardiology, Medical University of Vienna, 1090 Vienna, Austria; 2 Department of Pediatrics and Adolescent Medicine, Division of Pediatric cardiology, Medical University of Vienna, 1090 Vienna, Austria; 3 Department of Biomedical Imaging und Image guided Therapy, Medical University of Vienna, 1090 Vienna, Austria; 4 Department of Radiology, Ambulatorium Döbling, 1190 Vienna, Austria; 5 Department of Internal Medicine II, pulmology, Medical University of Vienna, 1090 Vienna, Austria

**Keywords:** systemic right ventricle, ccTGA, TGA, Mustard, Senning, atrial switch, 3D-Echocardiography

## Abstract

Background: The right ventricle serves as the subaortic systemic ventricle (sysRV) in patients with congenitally corrected transposition of the great arteries (ccTGA) and in patients with transposition of the great arteries (TGA) surgically repaired by an atrial switch. SysRV can lead to late complications, primarily heart failure, significant regurgitation of the systemic atrioventricular (AV) valve, and ventricular arrhythmias with sudden cardiac death. We sought to investigate the value of 2D- and 3D-echocardiographic parameters of sysRV function. Methods: Consecutive adult patients with sysRV who presented at the adult congenital heart disease outpatient clinic were prospectively enrolled. All patients received comprehensive transthoracic echocardiography, including 3D-echocardiography, cardiac magnetic-resonance-imaging (CMR), cardiopulmonary-exercise-testing, and blood analysis for NT-proBNP. Results. A total of 27 patients were included, 18 with TGA and nine with ccTGA. Median age was 37 years (Q1 = 31, Q3 = 44), 44% were male, median NT-proBNP was 189 pg/mL (Q1 = 155, Q3 = 467); sufficient 3D-echocardiography datasets were acquired in 78% of patients. All echocardiographic 2D and 3D volumetric function parameters correlated with CMR data, whereas a correlation was not seen with any of the longitudinal function parameters. NT-proBNP correlated with tricuspid annular plane systolic excursion (r = −0.43, *p* = 0.02) and CMR ejection fraction (EF) (r = −0.62, *p* = 0.003). Conclusion: Systematic evaluation of sysRV is complex and should include not only volumetric parameters but also parameters of longitudinal function in addition to measurement of NT-proBNP. In patients with good image quality, 3D-echocardiography can be used to assess volumes and EF.

## 1. Introduction

The right ventricle (RV) serves as the subaortic systemic ventricle in patients with congenitally corrected transposition of the great arteries (ccTGA) and in patients with transposition of the great arteries (TGA) surgically repaired by atrial switch. TGA occurs in 1:3100 live births and ccTGA in 1:33,000 live births [1]. Surgical methods for the repair of TGA by performing an atrial baffle repair were developed by Senning and Mustard in the 1950s and 1960s, respectively. This method includes complex surgery of the atria and leaves the RV in a systemic position [2,3]. Although the atrial switch procedure was replaced by arterial switch surgery in the early 1990s, patients who have undergone atrial switch and those with ccTGA still represent an important cohort in clinics following adults with congenital heart disease (ACHD).

An RV in the systemic position (sysRV) can lead to late complications, primarily heart failure, significant regurgitation of the systemic atrioventricular (AV) valve, and ventricular arrhythmias with sudden cardiac death [4,5,6,7]. Several studies have investigated morbidity and mortality in patients with sysRV. Prospective studies have demonstrated a cumulative survival of 60–77% after 30 years of follow-up [8,9].

As a result of chronic pressure overload, RV dilatation and RV hypertrophy are present in all patients with sysRV. Transthoracic echocardiography (TTE) is the first-line diagnostic technique used for the quantification of sysRV size and function. In patients with reduced RV function, mere visual classification of the degree of dysfunction is inaccurate even if performed by expert echocardiographers [10]. Therefore, a systematic evaluation of sysRV function must be performed to enable reliable follow-up.

The reference method for the assessment of RV volumetric function is cardiac magnetic resonance imaging (CMR). However, CMR is time-consuming, expensive, not universally available, cannot be used in some patients with claustrophobia and in some patients with cardiac pacemakers, particularly in those where the device was originally implanted shortly after surgery in the 1980s. True RV volumes can be measured by 3D echocardiography irrespective of the RV anatomy and geometry. A good correlation has been demonstrated between CMR-derived RVEF and 3D echocardiography-derived RVEF in several diseases, including ACHD [11,12,13], but to date, 3D echocardiography has not been studied in patients with sysRV. Therefore, the current study sought to investigate the value of commonly applied 2D and 3D echocardiographic parameters of RV function in patients with sysRV.

## 2. Methods

Consecutive adult patients with ccTGA or TGA surgically corrected by atrial switch who presented at the ACHD outpatient clinic between May 2017 and October 2018 were prospectively enrolled. All patients received comprehensive transthoracic echocardiography, including 3D echocardiography, CMR (unless contraindicated), cardiopulmonary exercise testing, and blood analysis for NT-proBNP.

All patients provided written informed consent and the study was approved by the local ethics committee (EK #1439/2017). The study protocol conformed to the ethical guidelines of the Declaration of Helsinki.

### 2.1. Echocardiography

Standard transthoracic echocardiograms (2D, Doppler, 3D) were obtained from all patients using echocardiography systems equipped with 3.5 MHz transducers (Vivid E9; General Electric (GE) Healthcare, Chicago, IL, United States). Echocardiography was performed in accordance with the recommendations of the American Society of Echocardiography and the European Association of Cardiovascular Imaging [14,15]. A data analysis was performed on a GE EchoPAC Clinical Workstation. 

**Figure 1:**
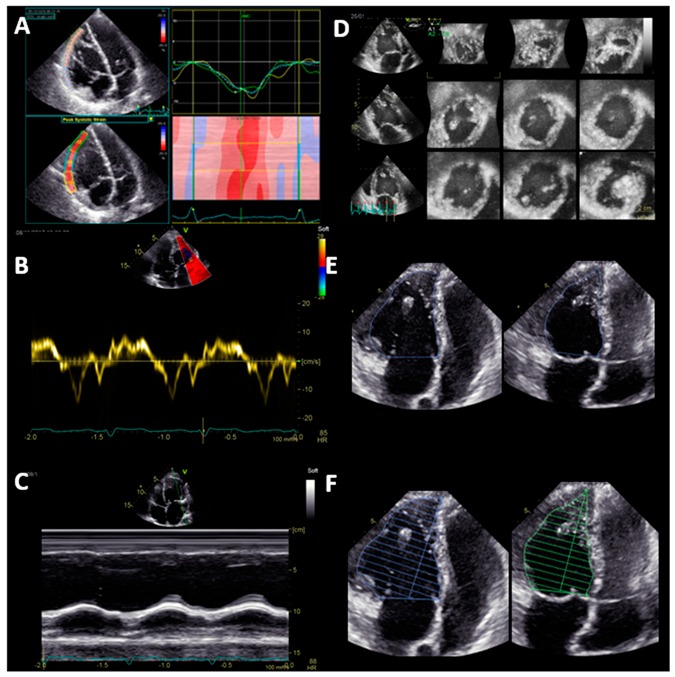
Systematic echocardiographic evaluation of systemic right ventricular function: Longitudinal function (Panel A-C) and volumetric function (Panel D-F). (**A**) Global longitudinal strain of the free lateral wall; (**B**) tissue Doppler velocity of the basal lateral wall S’; (**C**) tricuspid annular plane systolic excursion (TAPSE); (**D**) volumetric function assessed by 3D echocardiography; (**E**) fractional area change (FAC); (**F**) monoplane Simpson ejection fraction.

Two different aspects of sysRV function are reported: (1) volumetric function parameters, i.e., 3D end-diastolic volume (EDV), 3D end-systolic volume (ESV), 3D stroke volume (SV), 3D EF, 2D monoplane EF, 2D biplane EF, and FAC; and (2) longitudinal function parameters, i.e., GLS, TAPSE, and S’ (Figure 1).

The basal longitudinal function of the RV can be assessed by tissue Doppler imaging (S’) and by M-Mode. S’ is the peak systolic velocity of the tricuspid annulus measured by tissue Doppler imaging in an apical four-chamber view. For TAPSE, in an apical four-chamber view, the maximal systolic excursion of the lateral tricuspid annulus is measured by M-Mode. GLS of the free lateral wall of the RV was calculated by averaging the peak systolic strain of the three segments of the free lateral wall in an RV-focused four-chamber view. All 3D volumes and EF of the sysRV were analyzed both with TOMTEC 4D RV Function software (TOMTEC Imaging Systems, Munich, Germany) and with GE 4D Auto LVQ software (General Electric Healthcare, Chicago, IL, United States).

All datasets were recorded and analyzed by the same examiner (MS). Acquisition of a 3D echocardiography dataset was performed in all patients. Image quality was graded by one observer (MS) as sufficient or insufficient for analysis. Those graded sufficient were subdivided into the groups excellent, good, and moderate image quality. At the time of data analysis, the examiner was blinded to the CMR results. To assess intra-rater variability, seven randomly selected patients were analyzed a second time by the same observer. To determine inter-rater variability, seven randomly selected patients were analyzed by a second observer (MB).

### 2.2. Cardiac Magnetic Resonance Imaging

All CMR examinations were performed using a 1.5-Tesla Scanner (Magnetom Avanto Fit; Siemens Healthineers, Erlangen, Germany) with standard protocols. In order to assess RV volumes, an axial stack of cine data was acquired using balanced steady-state free precession imaging (repetition time msec/echo time msec, 3.2/1.2; flip angle, 64°; voxel size, 1,431,436 mm; 1,803,256 matrix). Trabeculations and papillary muscles were included as part of the RV volume [16]. To determine inter-rater variability, seven randomly selected patients were analyzed by a second observer (CN).

### 2.3. Cardiopulmonary Eexercise Testing

Cardiopulmonary exercise testing (CPET) was performed to assess maximal exercise capacity. The patients were placed on an exercise bicycle to perform continuous measurements of minute ventilation, oxygen consumption, heart rate, blood pressure, and electrocardiography. Workload was increased to achieve maximal effort in 10 min. Maximal peak oxygen consumption (VO_2_) and predicted maximal peak oxygen consumption (predVO_2_) are reported.

### 2.4. Laboratory Tests

In all patients, the cardiac biomarker NT-proBNP (normal value < 125 pg/mL) was analyzed from a blood sample drawn directly after completion of echocardiography according to the local laboratory’s standard procedure.

### 2.5. Statistical Analysis

Independent sample *t*-tests were used to analyze differences among groups. CMR and 3D echocardiography measurements of sysRV volumes, EF, and SV were compared using Pearson’s correlation coefficient. The Bland–Altman analysis was performed to measure the agreement among the methods for each parameter. Intra-observer and inter-observer variabilities were determined by intra-class correlation coefficients (ICC) using a two-way random model for degree of consistency. A *p*-value ≤ 0.05 was considered statistically significant. SPSS Version 24 (IBM SPSS, Armonk, NY, USA) was used for all analyses.

## 3. Results

### 3.1. Study Population

A total of 27 patients were included in the study, 18 with TGA (12 with Senning surgery, six with Mustard surgery) and nine with ccTGA. None of the ccTGA patients had significant associated lesions such as ventricular septum defect or outflow tract obstruction. The baseline characteristics of the study cohort are summarized in Table 1. The median age was 37 years (Q1 = 21, Q3 = 44), 44% were male, and the median NT-proBNP level was 189 pg/mL (Q1 = 155, Q3 = 467). A total of 81% of patients were New York heart association (NYHA) class I, and one patient was NYHA class III; the median VO_2_ on CPET was 22 mL/kg/min (Q1 = 17, Q3 = 25), median predicted VO_2_ on CPET was 55.9% (Q1 = 47.5, Q3 = 62). At least moderate regurgitation of the systemic AV valve was present in 44% of the ccTGA patients and in 22% of the TGA patients (*p* = 0.049).

### 3.2. Function of the Systemic Right Ventricle, 2D Echocardiography:

The sysRV was analyzed by different parameters of volumetric and longitudinal function. GLS, FAC, TAPSE, and 2D monoplane EF were measured in all patients; S’ and 2D biplane EF were measured in 24 patients. The median value was −14% for GLS; 0.09 m/s for S’; 13 mm for TAPSE; 28% for FAC; and 39% and 41% for mono- and biplane EF by 2D Simpson method, respectively. In 2D echocardiography, there were no differences between the two groups (ccTGA and TGA) regarding volumetric function and longitudinal function parameters (Table 1).

### 3.3. Function of the Systemic Right Ventricle, 3D Echocardiography

**Figure 2:**
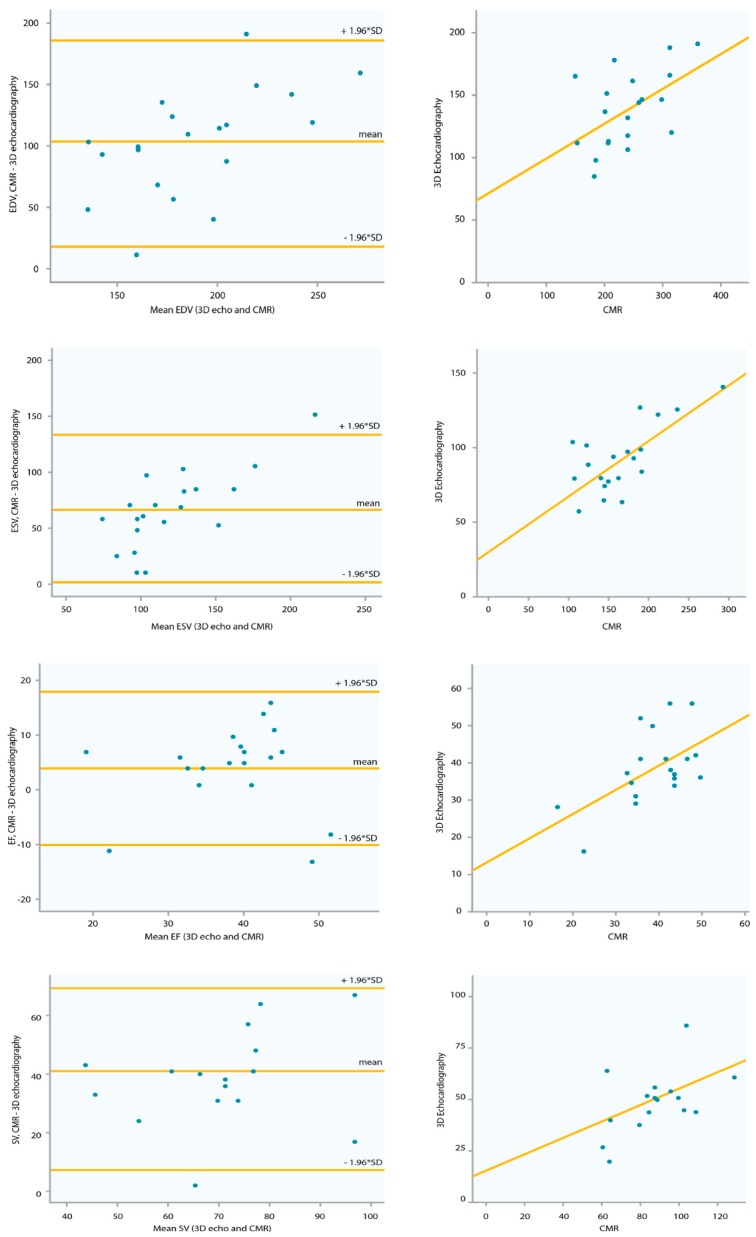
Correlation and Bland–Altman plots of 3D echocardiography and CMR data. CMR = cardiac magnetic resonance imaging. EDV = end-diastolic volume, ESV = end-systolic volume, SV = stroke volume, EF = ejection fraction, SD = standard deviation.

A sufficient 3D dataset of the sysRV was recorded in 21 (78%) of the patients. Among these, image quality was excellent or good in nine patients (43%) and moderate in 12 patients (57%). CMR was performed in 21 of the patients. All 2D and 3D volumetric function parameters correlated with CMR data, whereas none of the longitudinal function parameters correlated with CMR data (Table 2). Correlation between CMR and 3D echocardiography was acceptable regarding volumes and EF. Figure 2 shows the Bland–Altman analysis for the two methods in the overall group. The mean difference of EDV was 102 mL ± 43.44 mL. The mean difference of ESV was 64.9 mL ± 34.95. The mean difference of EF was 4% ± 7.49. The mean difference of SV was 38 mL ± 16.5. Volumes were lower in 3D echocardiography compared with CMR (Table 3).

Volumes derived from GE’s left ventricular 3D volume software showed no correlation with CMR values.

### 3.4. Inter/Intra-Observer Variability

Intra-rater and inter-rater variability were analyzed by ICC for the entire cohort and for those patients with good image quality. The inter-rater variability for 3D echocardiography was 0.84 (0.08–0.97), 0.88 (0.32–0.98), 0.74 (−0.51–0.96), and 0.63 (−1.14–0.94) for EDV, ESV, EF, and SV, respectively. Intra-rater variability was 0.64 (95% CI, 0.12–0.85), 0.71 (0.29–0.88), 0.85 (0.64–0.94), and 0.82 (0.58–0.93) for EDV, ESV, EF, and SV, respectively, for the overall group; and 0.82 (0.21–0.96), 0.78 (0.04–0.95), 0.82 (0.22–0.96), and 0.88 (0.48–0.97) for EDV, ESV, EF, and SV, respectively, for the subgroup of patients with good image quality. The inter-rater variability for CMR was 0.86 (−0.3–0.99), 0.96 (0.86–0.99), 0.88 (−0.17–0.99), and 0.88 (−0.17–0.99) for EDV, ESV, EF, and SV, respectively.

### 3.5. Exercise Capacity, NT-ProBNP

VO_2_ max did not correlate with any of the volumetric function or longitudinal function parameters. NT-proBNP correlated with CMR-EF (r = −0.62, *p* = 0.003) and TAPSE (r = −0.43, *p* = 0.02), and there was a trend toward correlation with 3D-Echo-EF (r = −0.37, *p* = 0.07) and GLS (r = 0.377, *p* = 0.053).

Two patients experienced major events during the study period. One ccTGA patient received a mechanical prosthetic systemic AV valve due to symptomatic severe regurgitation, and another suffered sudden cardiac death. Both were the only patients in this cohort with NT-proBNP values > 1000 (4400 and 1200, respectively).

## 4. Discussion

Systematic follow-up of patients with sysRV is required for the evaluation of different aspects of ventricular function. Echocardiographic examination should include assessment of both volumetric and longitudinal function. The current study shows that where adequate image quality can be obtained, 3D echocardiography is a feasible method to measure 3D volumes. Longitudinal function describes another aspect of ventricular function. NT-proBNP levels correlate with parameters of volumetric and longitudinal function.

While ventricular dilatation and a reduction in EF have been reported as the regular course for patients with sysRV, which sysRV volume parameters and EF values best reflect this situation remains the subject of some discussion. In the current study, median EDV measured by CMR was 240 mL in the overall group, and median EF was 41%. The correlation between CMR and 3D echocardiography was acceptable regarding volumes and EF, while the Bland–Altman plots showed a significant bias between the two methods in the overall group. In 33% of the patient cohort, 3D echocardiography datasets could be obtained with excellent or good image quality; this included patients with a variety of EF values (median 43%, Q1 = 32.5%, Q3 = 49%). If image quality was good, inter- and intra-rater variability were low. However, in the patients with inadequate image quality, inter-rater variability was high. As described in previous studies, volumes obtained by CMR were significantly higher than those obtained by echocardiography. Therefore, values measured by these two methods cannot be used interchangeably for follow-up [12,13].

Of the 2D volumetric parameters, FAC, monoplane EF, and biplane EF all showed a good degree of correlation with CMR-EF. It was possible to measure FAC and monoplane EF in all patients, and biplane EF was assessed in the majority of patients. Simpson’s method for volume calculation relies on geometric assumptions. This can be a significant pitfall especially when applied to the RV. Nevertheless, the method has previously been applied for RVEF calculation [17]. A reason for the good correlation in our data could be chronic geometric alterations of the RV due to longstanding pressure overload, leading to a RV cavity more similar to the physiologic geometry of a LV.

CMR is expensive and contraindicated in some patients due to old pacemaker leads and systems. The current study shows that 3D echocardiography can be applied in sysRV patients, although inferior image quality can limit the reliability of this approach. We therefore suggest that 3D echocardiography be applied where good image quality can be obtained; in all other patients, biplane or monoplane volumes and EF and/or FAC should be reported.

In addition to volumetric function parameters, the analysis of longitudinal function parameters has been proposed [1]. In agreement with previous studies, the current study showed no correlation between longitudinal function parameters and sysRV EF. At the same time, longitudinal function has prognostic power in patients with sysRV [18]. TAPSE, S’, and GLS were reduced in our patient cohort with no significant differences between the TGA and ccTGA patient sub-groups. Longitudinal function appears to describe an additional and independent aspect of sysRV function, which requires further investigation.

Interestingly, none of the evaluated echocardiographic parameters correlated with VO_2_. A possible explanation is the close range of measurements, with a median VO_2_ of 22 mL/min/kg (Q1 = 17, Q3 = 25), while CMR-EF showed a wide range of values (16–61%). Another explanation is that sysRV-EF as a single parameter is not sufficient to predict exercise capacity or prognosis in these patients. With a median of 189 pg/mL (Q1 = 155, Q3 = 467), NT-proBNP values were rather low despite large ventricles with reduced EF.

Progressive volume overload in a failing ventricle increases diastolic wall stress and stimulates an up-regulation of systemic and local neurohumoral activation. Thus, neurohormones are commonly measured as a marker of disease progression in patients with systolic left ventricular failure [19]. In the current study, NT-proBNP levels correlated both with the volumetric function parameter CMR-EF and with the longitudinal function parameter TAPSE. This could explain the prognostic value of NT-proBNP, and stresses the importance of such an analysis during follow-up in these patients. In this cohort, two patients experienced major events during the study period. Both patients were female and had ccTGA, longitudinal function was reduced with a mean GLS of −9.5%, mean TAPSE of −9.5 mm, and CMR derived sysRV-EF was 16% and 35%. Both were the only patients in this cohort with NT-proBNP values > 1000 (4400 and 1200, respectively). In line with this finding, a previous study by Popelová et al. described a high risk of death associated with NT-proBNP levels > 1000 mg/mL in patients with sysRV after atrial switch surgery [20]. Further research is required to evaluate the importance of NT-proBNP further, as this is an objective, quick, and inexpensive parameter.

In light of the data reported here, we suggest the regular follow-up of patients with sysRV to assess (1) volumetric function (EDV and EF), which should be performed regularly by CMR, unless contraindicated. CMR also allows for additional baffle function assessment. In the period between CMR assessments, 3D echocardiography volumes should be obtained where image quality is excellent or good, otherwise, biplane or monoplane EF and/or FAC should be reported. (2) In addition, parameters of longitudinal function (GLS, TAPSE, S’) should be obtained as these appear to provide an additional aspect of RV function. (3) Assessment of NT-proBNP levels can provide further insight into the clinical status of the patient, and it serves as an additional follow-up parameter that correlates with measures of volumetric and longitudinal function, as well as predicting mortality.

This study has a number of limitations. Firstly, the data only reflect the experience of a single tertiary care center, although this also ensures the inclusion of a homogenous patient population and consistent quality of imaging. Secondly, the study population is too small to allow subgroup analysis, e.g., sex-specific aspects. However, the sample size is similar or larger than previous studies of patients with sysRV [21].

## 5. Conclusions

Systematic evaluation of sysRV is complex and should include not only parameters of volumetric function but should be complemented by longitudinal function, in addition to markers of neurohumoral activation such as NT-proBNP. In patients where good image quality can be obtained, 3D echocardiography should be applied to report volumes and EF.

## Figures and Tables

**Table 1:** Baseline characteristics and 2D echocardiography data. NYHA = New York heart association, ACE-I = angiotensin-converting-enzyme inhibitor, sysRV = systemic right ventricle, GLS = global longitudinal strain, TAPSE = tricuspid annular plane systolic excursion, AVV = atrio-ventricular valve, TGA = transposition of the great arteries, ccTGA = concentially-corrected transposition of the great arteries.

	Total	TGA	ccTGA	*p*-Value
N Baseline characteristics	27	18	9	
Age, years (median, Q1;Q3)	37 (31;44)	36 (31;41)	42 (29;52)	0.267
Male sex, n (%)	12 (44)	8 (44)	4 (44)	1
proBNP, pg/mL (median, Q1;Q3)	189 (155;467)	189 (156;444)	184 (105;855)	0.315
VO2max in CPET, mL/kg/min (median, Q1;Q3)	22 (17;25)	22 (17;24)	24 (16;24)	0.879
Predicted VO2max in CPET, % (median, Q1;Q3)	55.9 (47.5;62)	54.6 (48.2;66.7)	58.8 (40.3;60.4)	0.61
NYHA Class				0.523
I	22 (81)	15 (83)	7 (78)	
II	4 (15)	3 (17)	1 (11)	
III	1 (4)	0	1 (11)	
IV	0	0	0	
Medication				
Betablocker	8 (30)	3 (17)	5 (56)	0.072
ACE-I	17 (63)	11 (61)	6 (67)	0.789
Aldosterone antagonist	3 (11)	1 (6)	2 (22)	0.313
**2D Echo: sysRV function**				
GLS (%)	−14 (−19;−11)	−14.5 (−19;−11)	−14 (−20;−11)	0.894
S’ (m/s)	0.09 (0.08;0.11)	0.09 (0.07;0.11)	0.09 (0.08;0.11)	0.385
TAPSE (mm)	13 (11;16)	13.5 (11;15)	13 (11.5;16.5)	0.597
Fractional area change (%)	28 (25;34)	28 (24;34)	28 (22;32)	0.471
SysRV-EF monoplane Simpson (%)	39 (34;47)	38 (29;42)	40 (34.5;50.5)	0.23
sysRV-EF biplane Simpson (%)	41 (37;48)	41 (37;45)	42 (34;49.5)	0.809
**2D Echo: Valvular disease**				
Syst. AVV regurgitation				**0.049**
Mild	19 (70)	14 (78)	5 (56)	
Moderate	5 (18)	3 (17)	2 (22)	
Severe	3 (11)	1 (5)	2 (22)	

**Table 2:** Correlation of cardiac magnetic resonance imaging derived systemic ventricle ejection fraction with 2D echocardiographic parameters of longitudinal and volumetric systolic function. FAC = fractional area change, EF = ejection fraction, TAPSE = tricuspid annular plane systolic excursion, S’ = tissue Doppler velocity of the basal lateral wall, GLS = global longitudinal strain.

	Correlation R Value	*p*-Value
**Volumetric function**		
FAC	0.55	**0.01**
Monoplane EF	0.48	**0.03**
Biplane EF	0.65	**0.003**
**Longitudinal function**		
TAPSE	0.33	0.14
S’	0.17	0.49
GLS	−0.4	0.07

**Table 3:** Systemic right ventricular volumes determined by two different 3D echocardiography software solutions (tomtec and GE), as well as volumes as measured by cardiac magnetic resonance imaging. EDV = end-diastolic volume, ESV = end-systolic volume, EF = ejection fraction, SV = stroke volume, CMR = cardiac magnetic resonance imaging, TGA = transposition of the great arteries, ccTGA = congenitally corrected transposition of the great arteries.

3D Echo (Tomtec Software)	Total	TGA	ccTGA	*p*-Value
EDV, median (Q1;Q3)	146 (115;166)	141 (112;153)	162 (130;191)	0.096
ESV, median (Q1;Q3)	87 (70;98)	78 (70;95)	94 (66;117)	0.393
EF, median (Q1;Q3)	40 (34;49)	39 (35;41)	46 (31;54)	0.328
SV, median (Q1;Q3)	53 (34;49)	49 (42;57)	63 (52;85)	0.082
**3D Echo (GE 3D-LV software)**				
EDV, median (Q1;Q3)	162 (140;192)	150 (118;193)	165 (149;197)	0.657
ESV, median (Q1;Q3)	83 (66;100)	86 (61;105)	78 (66;91)	0.569
EF, median (Q1;Q3)	47 (36;51)	46 (37;51)	50 (35;54)	0.737
SV, median (Q1;Q3)	80 (58;90)	73 (58;87)	85 (66;94)	0.455
**CMR**				
EDV, median (Q1;Q3)	240 (205;280)	218 (204;266)	255 (227;318)	0.212
ESV, median (Q1;Q3)	141 (106;177)	125 (105;169)	168 (132;207)	0.233
EF, median (Q1;Q3)	41 (34;44)	41 (35;46)	38 (28;44)	0.352
SV, median (Q1;Q3)	89 (70;103)	89 (65;101)	89 (83;117)	0.251
